# Boosting Biomedical Research Capacity: The Role of INBRE-Supported Core Facility Vouchers in IDeA States

**DOI:** 10.7171/001c.159214

**Published:** 2026-06-01

**Authors:** Laura M. Lessard, Melinda K. Duncan

**Affiliations:** 1 Delaware INBRE University of Delaware; 2 Department of Health Behavior and Nutrition Sciences University of Delaware; 3 Department of Biological Sciences University of Delaware

**Keywords:** Core Facilities, Research Infrastructure, Voucher Programs, Capacity Building, Financing

## Abstract

Research universities and institutes invest in building core facilities that allow investigators to access the costly instrumentation and specialized expertise needed to perform cutting edge biomedical research. For the past 30 years, the National Institutes of Health has supported the Institutional Development Award (IDeA) initiative which supports research capacity, including core facility construction, in U.S. states which receive disproportionately little NIH funding. IDeA Network of Biomedical Research Excellence (INBRE) programs particularly support research in primarily undergraduate institutions (PUI) and other resource constrained sites, and have built core facilities intended to broaden access to biomedical research training to students enrolled at PUIs. However, researchers in IDeA states (especially those working at PUIs), often lack the funding needed to pay core facility user fees needed to collect preliminary data for grant applications or key experiments needed for publications, which reduces investigator competitiveness for future funding. In order to overcome this hurdle, INBRE programs serving 12 different states have developed voucher programs that support core facility access. Here we describe the structure of these programs and provide evidence from one state (Delaware) that these modest investments have a significant impact on research productivity as measured by downstream publications and grants, while also fostering the viability of research core facilities by building demand for core services. Across the country, INBRE-supported Core Voucher programs contribute significantly to the biomedical research enterprise fueling future research and innovation.

## Introduction

Technologies such as next gen sequencing, confocal microscopy, and mass spectrometry have revolutionized biomedical research. However, the instruments needed for this work are expensive and difficult to maintain while requiring specialized expertise to use; as a result, they are often not accessible to individual investigators. Core facilities are centralized research infrastructures that provide investigator access to cutting edge technologies while sharing instrument access and scientific expertise across multiple laboratories, which ensures that these costly resources are used efficiently.^1^ As such, core facilities are critical research infrastructure and are often supported as such; one group estimated that the overall annual investment in core facilities from the National Institutes of Health (NIH) alone is over $900 million.^2^ Despite the benefits that accrue from placing research resources within fee for service research core facilities, there remain challenges blocking core facilities from achieving their full potential as key tools supporting biomedical research innovation across the nation.

Core facilities are typically established by research institutions in response to an investigative need for a particular technology. Facility startups are often primarily funded by institutional funds (donations/indirect cost recovery), which cover renovations/core facility staff salary while major instrumentation and research enhancement grants populate the cores with needed equipment.

Funding is a major barrier to high quality service provisions among core facilities and can have wide ranging impacts on both the facility itself and the potential scientific results.^3^ Longer term, core facilities must have consistent funding to ensure a sustainable investment plan for acquiring, upgrading, and maintaining expensive equipment, including budgeting for maintenance contracts, software tools, data storage, and computing capacity.^1^ Furthermore, the most impactful core facilities also invest in maintaining a staff that are experts in the hosted technologies to ensure user training in appropriate experimental design and equipment operation. Core facility operating budgets in the United States are typically supported by core facility user fees, which can be augmented by the institution’s indirect cost recovery or other funding sources as some cutting edge technologies (i.e., magnetic resonance imaging, spatial transcriptomics, flow cytometry, etc.) require costly maintenance and highly trained staff support that would make full cost user fees cost prohibitive to some academic researchers that are seeking major funding and/or working in resource limited environments such as primarily undergraduate institutions (PUIs). These financial challenges contribute to academic researchers experiencing uneven access to core facility resources based on geography and institutional wealth, which limits their ability to use the most appropriate technology to answer their research questions while training young scientists in cutting edge methods.^1^ These challenges led to a gathering of the Federation of American Societies for Experimental Biology (FASEB) concluding that, “large majority of institutions of higher education do not have access to [core] facilities.” They further suggested that expanding equitable access is needed.^4^

However, access to core facilities across academic institutions is challenging as core facility budgets are typically highly subsidized by institutional resources leading institutions (or even departments/colleges within institutions) to exclusively limit access to those working at the entity hosting the core facility, or only allow access to users from outside of the institution if they are able to pay higher “outside” user fees to recoup the institutional investments made by the host institution. Thus, researchers at universities with less research activity are often “locked out” from core facility access because their home institution lacks the ability to subsidize a core facility while access to core facilities at neighboring institutions is either blocked or cost prohibitive.

The NIH-funded IDeA provides funding to build biomedical research facilities in 23 states and Puerto Rico, areas that historically have had lower levels of NIH funding. IDeA Networks of Biomedical Research Excellence (INBRE) programs are statewide initiatives to support faculty research at under-resourced institutions (predominantly undergraduate institutions), training of undergraduates in biomedical research, and data science/instrumentation cores that are accessible to all researchers within the state. While IDeA program investments have enhanced the availability of accessible research core facilities to scientists working in IDeA states, it is still difficult for researchers working at under-resourced institutions to gain access to the research instrumentation needed to train undergraduates in cutting edge research methods and generate the preliminary data needed to gain independent research funding (such as the NIH R15/R16 program for scientists at teaching intensive institution) due to a lack of funding to pay user fees. One potential solution to this challenge is to create a mechanism by which faculty can apply for small scale vouchers to cover core facility fees. These vouchers can also facilitate conversations with core facility staff that can generate ideas for future research using emerging technologies as staff possess technical expertise that can combine with investigator knowledge.^5^ One institution reported that a core facility voucher program was well received by both investigators and core directors, and data from these programs can be used for planning purposes within institutions and cores.^6^ Another voucher program that was implemented across a state that receives substantial federal funding reported that vouchers distributed from 2009 to 2019 contributed to 220 peer-reviewed publications and 225 funded grants.^7^ Despite these promising results, there is little direct evidence that implementing voucher programs in IDeA states (e.g., those with limited research infrastructure or NIH-funded research activity) is effective in increasing research activity. This article tabulates the INBRE programs that offer vouchers or similar programs to foster core facility access across their state and highlights the impact of the Delaware INBRE voucher program to improve research activity across Delaware.

## Methods

The principal investigators of all INBRE programs active during 2023 and 2024 were contacted and asked to share 1) whether they used voucher programs to foster core facility access within their state; and 2) if so, what is the structure of their program. That process identified 12 INBRE programs and 1 regional collaboration of 6 INBRE programs that had active core facility voucher programs. Evaluation data from these programs were collected and aggregated, including data on program description, applicant eligibility, specifics of which facilities could be accessed, and data on the total number and dollar amount of vouchers funded. Given the diversity of the programs and the time periods for which data was available, data summarizing common data elements are presented.

Delaware INBRE also collected quantitative data from 12 recipients who received vouchers in 2022 and 2023 and 23 recipients of vouchers in 2023 and 2024. Interview questions focused on recipients’ experiences with the voucher program along with the impact of their voucher on their research program and productivity. Interviews were recorded, transcribed, and subsequently analyzed using thematic analysis to identify commonalities. Voucher recipients from 2023–2024 were sent an online survey asking about the impact of the voucher, including details about publications, grants and presentations. The data were cleaned and aggregated.

## Results

### Program Descriptions

Of the 24 NIH-funded INBRE programs, 18 offer investigators within their state the ability to apply for vouchers to cover the cost of core facility access, although there is a wide variability between the name, structure, and focus of these programs (Table 1). Twelve of these INBRE voucher programs have a statewide focus while a consortium of western states (RAIN- Alaska, Hawaii, Idaho, Montana, Nevada, New Mexico, Wyoming) collaborate to foster core facility access across the region. While all voucher programs are open to faculty and other independent researchers, four jurisdictions (AR, ME, NV, PR) also allow students to apply to the program.

**Table 1.** Summary of INBRE-supported core facility voucher programs eligibility and use

**State(s)**	**Program name**	**Eligibility**	**Funds available**	**Eligible facilities**
Alaska	Bioinformatics Service Awards	UA faculty member, UA postdoc, or partner institution researcher	$10,000	Bioinformatics
Arkansas	Core Facility Voucher Program	Faculty and student researchers	$7,500	UAMS and UAF Core Facilities
Delaware	Core Facility Voucher Program	Faculty and researchers across the state	4,000percore;upto8,000 for multicore	A range of 22 facilities including bioinformatics, imaging, and data science
Idaho	Technology Access Grant	Faculty investigators	Range from 5,000to10,000. Amounts up to $15,000 if justified based on requirements	IBEST Genomics Resources Core, Computational Resources Core, and Optical Imaging Core
Kentucky	Electron Microscopy Voucher Program	PUI researchers and UK and UofL	$5,000	Facilities at the University of Louisville or University of Kentucky
Louisiana	Core Bucks	Project investigators	$1,500	Range of institutions
Maine	Core Use Access Grants	Faculty and students across the state	$5,000 per award	Facilities across the state and within the northeast region
Mississippi	Award Supplement	Staff and faculty		Full-service core at University of Southern Mississippi; Molecular Genomics Core Facility at University of Mississippi Medical Center; Proteomics Core Facility at Mississippi State University
Nevada	Core Service award program	Faculty and Students from any discipline	$5,000 max	Nevada Bioinformatics Center at UNR, Mick Hitchcock Proteomics Center at UNR, UNVL Genomics Core Facility, Core Analytical Lab at UNR, Nevada Genomics Center
Puerto Rico	Feasibility Awards	Faculty and students	5,000−15,000	Any COBRE in Puerto Rico
Rhode Island	Bioinformatics Pilot Project (Molecular Informatics Core)	Tenure-track assistant or associate professors at Rhode Island PUIs	$10,000 max	Any Rhode Island Primarily Undergraduate Institution
RAIN states (Alaska, Hawaii, Idaho, Montana, Nevada, New Mexico, Wyoming)	Tech Access program	Any Project Investigator from an institution located within the network of a RAIN state	2,000−5,000	

There was a range of available funding options, with the lowest value at $1,500 in Louisiana ranging to up to $15,000 in Idaho and Puerto Rico. This variation can be partially attributed to the diversity of cores that are accessed through these programs; some vouchers were limited to a single facility or a small number of facilities while others were open to a broader list of facilities that may have substantial cost variation. For example, the Alaska voucher program is limited to accessing the Bioinformatics Core while the Core Use Access Grants from Maine INBRE can be used to access facilities across Maine and the entire northeast region.

### Impact: Awards and Funds

Across the programs with available data, 919 vouchers worth over $2.9 million in total funding were reported (Table 2). Due to the nature of the available data, these awards were made over different time spans with some stretching back to as early as 2015.

**Table 2.** Award information for INBRE-supported core facility voucher programs

**State**	**Program name**	**Number of awards (time period)**	**Total funds awarded**
Alaska	Bioinformatics Service Awards	37 (2014–2023)	$205,588
Arkansas	Core Facility Voucher Program	206 (2015–2023)	$501,000
Delaware	Core Facility Voucher Program	105 (2019–2022)	$521,358
RAIN	Technology Access grant	82 (2018–2024)	$305,537
Idaho	Feasibility Award	21 (2015–2023)	$129,698
Kentucky	Electron Microscopy Voucher Program	33 (2020–2024)	$165,000
Louisiana	Core Bucks	Unavailable	Unavailable
Maine	Core Use Access Grants	16 (2019–2024)	$80,000
Mississippi	Award supplement	23 (2017–2023)	$43,585*
Nevada	Core Service award program	289 (2018–2023)	$531,429.96
Puerto Rico	Feasibility awards	21 (2015–2023)	$129,698
Rhode Island	Bioinformatics Pilot Project (Molecular Informatics Core)	4 (2021–2024)	$17,630.02
RAIN states	Tech Access program	82 (2019–2023)	$305,537
	**Totals**	**919 awards**	**$2,936,061**

* This amount represents total costs of 5 of the 23 uses under this program. Total amounts for the remaining uses are not available.

### Impact: Evidence from Delaware

Delaware INBRE has collected qualitative and quantitative data on the impact of their Core Voucher program, which offers access to more than 19 different cores located at 4 different Delaware institutions. During the period from May 1, 2023, to April 30, 2025, approximately 89 vouchers were awarded across the state. The Bio-Imaging Center at the University of Delaware was the most utilized core with 23 vouchers. Other frequently used cores include the DNA Sequencing & Genotyping Center and the Flow Cytometry Core (both at the University of Delaware), each with 10 vouchers. The Bioinformatics Data Science Core and the Mass Spectrometry Core at the University of Delaware also saw significant use with 9 and 8 vouchers, respectively. The 89 vouchers were distributed broadly across scientific disciplines, but there was a concentration of vouchers for neuroscience and cancer research. Neuroscience was the largest category of funded vouchers, covering everything from molecular neuroscience to complex behaviors.

Data from interviews with voucher recipients suggest that the program positively impacts productivity via grants and manuscripts, and they can facilitate the generation of new research ideas. Several interviewees used the data collected to support new grant applications or to address weaknesses in their previous grant submissions. Other recipients used the data to generate manuscripts and other reports. Using quantitative data from 23 voucher recipients from the period of May 1, 2023, to April 30, 2024, the vouchers accounted for 10 manuscripts, 12 grant submissions (2 have already been funded), and 25 presentations at scientific conferences and meetings.

The most commonly reported impact from interviews was more broadly focused on the research development process, giving investigators the flexibility to pursue new directions or explore new equipment. According to one recipient, “While working on an NIH-funded project, the research team decided to go in a new direction that required funding to access equipment. The voucher allowed us to explore new methods that will contribute to the development of our project.” Interviewees credited the combination of the data generated from the core and the expertise provided by core facility staff, suggesting that access alone is only one component of the voucher programs. One recipient credited core facility staff for helping them see that an initial research approach was not feasible while helping them identify alternative pathways for answering their research questions.

While there are programs to support the development and operations of core facilities, another challenge is the lack of knowledge of which facilities exist and where they are located.^2^ Facilities need support to effectively communicate the facility’s capabilities to scientists, build collaborations with industry partners, and establish cross-institutional alliances to share expertise and resources.^1^ In IDeA designated states, INBRE programs often take the lead in coordinating and communicating the capabilities of core facilities in their states by sharing this information with students and investigators across their networks.

## Discussion

These data suggest that the INBRE program at large provides substantial and meaningful access to core facilities across our jurisdictions. The variation in eligibility across states suggests that these programs can also be flexible to meet the needs within an individual context. For example, existing institutional resources may be available to facilitate student use, while externally funded Core Voucher programs can focus on investigators and others without existing support. Leveraging existing support and filling gaps is one major broad goal of the INBRE programs, and the voucher programs illustrate that approach.

The evidence from the Delaware voucher program suggests that even these modest investments can have substantial impacts on research productivity. They facilitated the pursuit of new research ideas, which may be a particularly powerful opportunity for Core Voucher programs as some of this innovation takes place during the grant writing or application process when traditional funding is not yet available. While these types of data were not available for other jurisdictions, the Delaware program operates similarly to other states; thus, it is likely that a similar impact could be expected from other INBRE programs’ vouchers.

Future evaluation and research should continue to document the impact of these voucher programs on downstream research outputs such as publications, presentations, and grants. That information could be particularly useful when advocating for the creation or continuation of voucher programs within institutions and networks. Previous research has leveraged core facility management software to explore these patterns,^6^ which could be a powerful data source for future research.

While our data focus is on INBRE programs, there are other sources of core facility access funding across the country. For example, the CTSA program also provides support for core facilities; however, the majority of these awards are located in non-IDeA states.^8^ The NIH IDeA-funded Centers of Biomedical Research Excellence (COBRE) program invests in the development and support of research cores, and recent research suggests that these facilities are heavily used by investigators at COBRE institutions.^9^ In sum, these types of programs have the potential to significantly improve access to biomedical research facilities and continue to expand scientific discoveries into the future.

## Data Availability

Not applicable.
